# Effect of Annealing on the Electrical and Magnetic Properties of Ni-SiO_2_ Multilayer Nanocomposites

**DOI:** 10.3390/nano16161033

**Published:** 2026-08-20

**Authors:** Aleksandra Wilczyńska, Mateusz Łakomski, Łukasz Ruta

**Affiliations:** 1 Department of Electronics and Information Technology, Lublin University of Technology, 38A Nadbystrzycka, 20-618 Lublin, Poland; 2 Department of Semiconductor and Optoelectronic Devices, Lodz University of Technology, 8 Politechniki Ave, 93-590 Lodz, Poland; mateusz.lakomski@p.lodz.pl (M.Ł.); lukasz.ruta@p.lodz.pl (Ł.R.)

**Keywords:** nanocomposites, AC parameters, magnetic parameters, structural and chemical analysis, computer simulations

## Abstract

This study investigates the influence of annealing on the electrical, magnetoresistive, and structural properties of Ni–SiO_2_ nanocomposites fabricated by magnetron sputtering. Electrical measurements performed over the frequency range of 4 Hz to 1 MHz and at temperatures between 298 and 333 K revealed that charge transport in the structures is dominated by hopping conduction described by the Mott and Jonscher models. Before annealing, the nanocomposite exhibited behavior characteristic of a system near the percolation threshold, while thermal treatment at 673 K for 30 min significantly reduced conductivity and increased activation energy, indicating reorganization of conductive pathways and increased separation between active centers. Phase angle analysis confirmed the coexistence of resistive and capacitive components, associated with Maxwell–Wagner–Sillars interfacial polarization. Magnetoresistance measurements demonstrated a transition from negative magnetoresistance in the non-annealed sample to positive magnetoresistance after annealing, suggesting a change from spin-dependent scattering to tunneling-dominated transport mechanisms. Electric and magnetic field simulations were carried out, which showed that the magnetic field was uniformly distributed throughout the structure. SEM observations confirmed a granular morphology with increased intergrain separation after annealing, EDS analysis indicated no significant increase in oxidation. The obtained results demonstrate that annealing strongly modifies transport and magnetic properties of Ni–SiO_2_ nanocomposites, making them promising materials for magnetoelectronic applications.

## 1. Introduction

In contemporary materials science and nanoelectronics, one of the greatest challenges is creating materials that combine seemingly contradictory physical properties. The answer to this need is provided by metal–dielectric nanocomposites, composed of nanometer-sized metallic structures, e.g., grains or thin layers, dispersed in an insulating oxide or ceramic matrix [1,2]. The combination of a conductive and magnetic phase with an insulating phase on the nanometer scale creates a completely new physical quality. Interface phenomena occurring at the interfaces and quantum mechanical effects, such as charge tunneling, are crucial here. By controlling the geometry of such a system, it is possible to control its electrical conductivity, dielectric permittivity, and magnetic properties [3,4,5]. Recent years have seen a rapid expansion in research on metal–dielectric nanocomposites, particularly multilayer structures containing ferromagnetic nanoparticles embedded in an insulating matrix. These systems exhibit compelling electrical, magnetic, and magnetoelectric properties, making them promising materials for applications in electronics, magnetic sensors, high-frequency systems, and memory components. Particularly noteworthy are nanocomposites based on nickel and silicon dioxide, which enable controlled charge transport, tunneling effects, and impedance changes under the influence of an external magnetic field [6,7,8,9,10]. Unlike classical random composites, multilayer structures offer precise control over the geometry of the system and the nature of the interfaces, enabling the engineering of transport barriers and the study of conduction anisotropy. In such systems, processes occurring at the interfaces play a significant role, including Maxwell–Wagner interfacial polarization and tunneling through dielectric layers [11,12].

The combination of ferromagnetic nickel with amorphous SiO_2_ provides a model system for studying the correlation between structure and charge transport in the presence of an external magnetic field. Previous literature analyses have focused primarily on granular composites. However, the transition to multilayer geometry opens new research opportunities, enabling precise control of the potential barrier thickness and reducing the effects of chaotic percolation, which often limits the repeatability of granular structures [13,14,15,16]. Multilayer Ni–SiO_2_ structures are characterized by a complex microstructure in which conductive metallic layers are separated by a high-resistivity dielectric. This structure favors the occurrence of hopping conduction mechanisms, interfacial polarization, and electron localization effects. These properties strongly depend on layer thickness, the number of layer repetitions, and the technological parameters of the fabrication process, such as magnetron sputtering conditions and heat treatment. Studies conducted on similar metal–SiO_2_ structures have shown that analysis of AC parameters allows for the determination of dominant charge transport mechanisms and modeling of equivalent electrical circuits describing the behavior of the nanocomposite [15,17].

Magnetron sputtering is one of the most commonly used methods for deposition of thin films and multilayer structures. This technique involves sputtering material from a cathode target in a working gas atmosphere, typically argon, while simultaneously utilizing a magnetic field to enhance plasma ionization. This allows for the production of uniform layers of controlled thickness, high purity, and good adhesion to the substrate. Thanks to the ability to precisely control process parameters, magnetron sputtering is widely used in the production of metal–dielectric nanocomposites [5,18,19]. Impedance spectroscopy is one of the fundamental methods for studying the electrical properties of nanocomposites. It allows analysis of the material’s response as a function of frequency and determination of parameters such as resistance, capacitance, and dielectric losses. This method also allows for the assessment of the influence of interphase boundaries and dielectric layers on charge transport [20]. Another important aspect of research on ferromagnetic nanocomposites is the effect of an external magnetic field on their electrical properties. The presence of a magnetic field can lead to changes in the material’s impedance due to spin interactions, changes in electron free path length, and magnetoimpedance phenomena [21,22].

Metal–dielectric nanocomposites currently constitute an important group of functional materials used in nanoelectronics and materials engineering. As a result, metal–dielectric nanocomposites find applications in spintronics and tunnel magnetoresistance (TMR), MRAM memories, memristors and neuromorphic systems, as well as electromagnetic shielding (EMI) materials and microwave absorbers used in high-frequency technologies [6,23,24]. Furthermore, the plasmonic properties of metallic nanostructures enable the use of such systems in optoelectronics, photonics, and biosensors [22,25,26,27,28,29,30].

## 2. Materials and Methods

### 2.1. Preparation of Multilayer Ni-SiO_2_ Nanocomposites by Magnetron Sputtering

Before the sputtering process, n-type silicon wafers with crystallographic orientation (110) were subjected to a multi-step chemical cleaning procedure to remove the natural oxide layer and organic and metallic contaminants from the surface. The substrate preparation process involved cutting the silicon wafers into samples of appropriate dimensions and then sequentially etching them in appropriately selected chemical mixtures. In the first step, the samples were etched for 10 min at 75–80 °C in a mixture of H_2_O_2_ (30%) and H_2_SO_4_ (95%) (1:2 volume ratio), which allowed for the removal of organic contaminants. The wafers were then rinsed with deionized water and subjected to another etching for 10 min at 75–80 °C in a mixture of H_2_O, NH_3_∙H_2_O (25%), and H_2_O_2_ (30%) (5:1:1 volume ratio). After another rinse with deionized water, the samples were etched for 10 min at 75–80 °C in a 6:1:1 volume mixture of H_2_O, HCl (35%), and H_2_O_2_ (30%) to remove metallic contaminants. The final cleaning step involved etching at room temperature for 10 min in a 1:50 volume solution of HF (40%) and H_2_O to remove the silicon oxide layer from the substrate surface.

**Figure 1:**
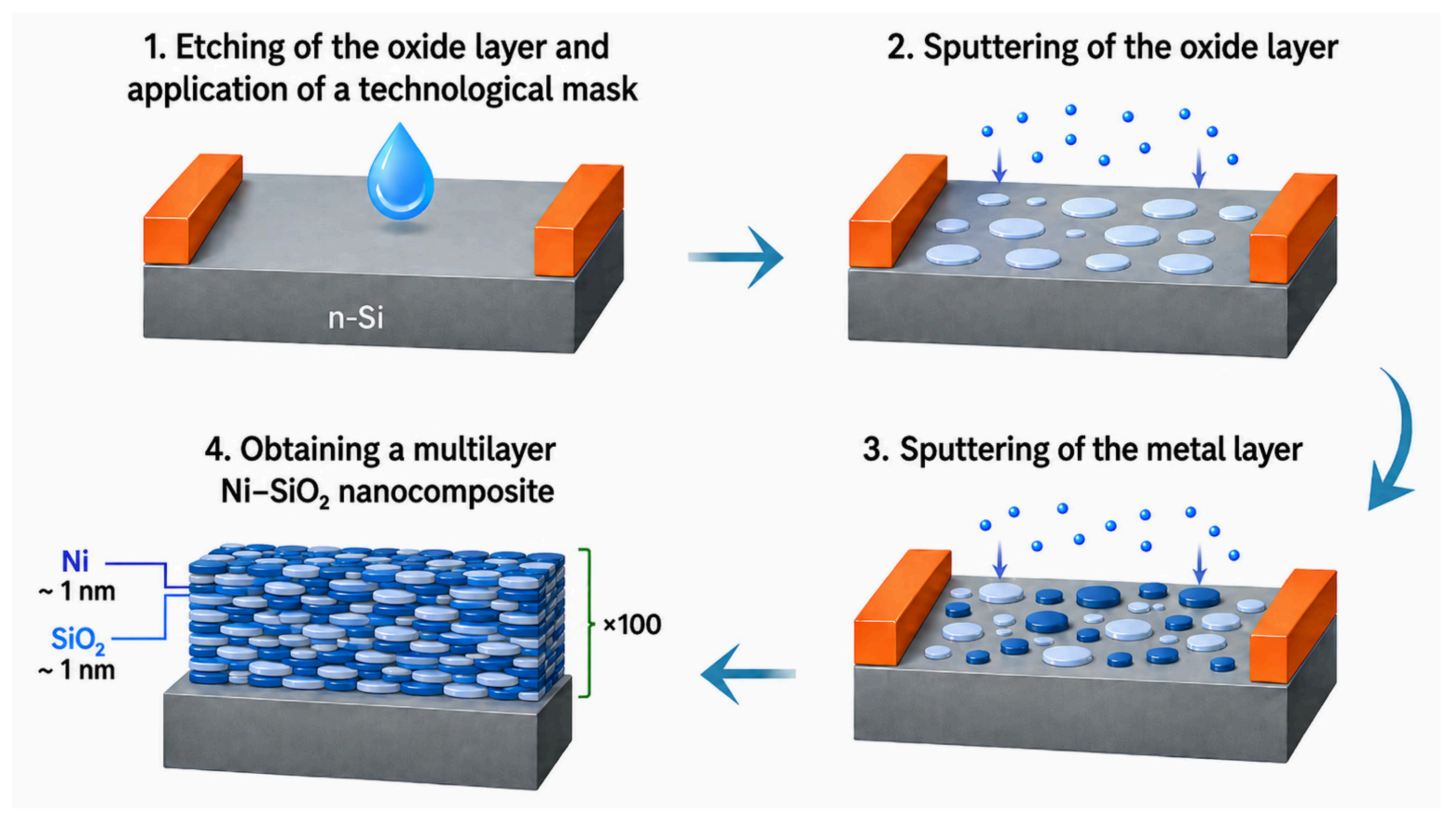
Technological processes carried out to obtain the multilayer Ni-SiO_2_ nanocomposite.

Thin-film deposition was performed using a dual-source magnetron sputtering method using a Moorfield MiniLab 026 (Moorfield, Knutsford, UK) sputtering device equipped with an Inficon SQM160 (Inficon, Bad Ragaz, Switzerland) thickness and speed measurement system. The process was conducted in an argon atmosphere with a working gas flow of 40 sccm. The operating chamber pressure during sputtering was maintained at 27 × 10^−3^ mbar. Two targets were used in the process: a 125 µm thick nickel (Ni) target and a silicon dioxide (SiO_2_) target (Kurt J. Lesker, Jefferson Hills, PA, USA). The Ni target was mounted on a DC magnetron, whereas the SiO_2_ target was mounted on an RF magnetron, with sputtering powers of 100 W and 20 W, respectively. Deposition was performed alternately, yielding a multilayer structure consisting of 50 nickel layers and 50 SiO_2_ layers, each with a thickness of approximately 1 nm; this thickness does not provide a continuous layer. During the process, eight samples were made and their values were comparable, confirming the reproducibility of the sputtering process. Figure 1 shows a diagram of the sputtering process.

Metal–dielectric structures are often described using percolation models, which are used to explain the insulator–conductor transition in composites containing conductive fillers in an insulating matrix. When the conductive phase fraction *x* is below the percolation threshold xc, charge transport occurs primarily through electron tunneling between metal grains and their thermally activated hopping [31]. After reaching a critical value (*x* = *x_c_*), the system enters a conducting state, and near it, a metal–insulator transition (MIT) is observed, which strongly influences the electrical properties of the nanocomposites. In the regime near the percolation threshold, the correlation length increases rapidly, which is associated with the formation of a network of interconnected metallic grains. In this range, the system exhibits distinctly different properties and increased sensitivity to electromagnetic fields [32].

### 2.2. Annealing of Structures and Measurements of AC Parameters of Ni-SiO_2_ Nanocomposites

Impedance spectroscopy is an advanced, non-destructive analytical method used to study the electrical properties of materials and processes occurring at phase boundaries. This technique enables the separation and quantitative analysis of various phenomena occurring in a system, including the kinetics of electrochemical reactions, diffusion processes, and the specific resistance of a material. Unlike methods based on direct current, in EIS, the system is excited by a small sinusoidal voltage signal. Measurements are performed over a wide frequency range, allowing for detailed analysis of the system’s response over various time scales [28].

Granular or multilayer nanocomposites have a disordered structure composed of a metallic phase *x* and a dielectric phase. As the conductive phase content approaches the percolation threshold *x_c_*, interconnected metal agglomerates begin to form in the material, forming current-carrying paths. For *x* < *x_c_*, charge transport occurs primarily through electron tunneling between grains separated by a dielectric, often accompanied by a negative temperature coefficient of resistance [33,34]. This mechanism results from the quantum penetration of the electron wave function through a potential barrier. In systems with a predominant dielectric phase, conductivity strongly depends on temperature and is described by the Mott variable-range hopping conductivity model described in Equation (1) [35]:

(1) $$\sigma \sim exp\left[-{\left(\frac{T}{T_{0}}\right)}^{-1/4}\right]$$

where *σ* denotes conductivity, *T* denotes temperature, and *T*_0_ denotes density of states at the Fermi level.

Electrical properties of the obtained structures were measured using a Hioki 3536 RLC meter. The tests were performed over a wide frequency range from 4 Hz to 1 MHz, enabling analysis of the electrical response of the samples at both low and high signal frequencies. The measurements were performed over a temperature range from room temperature to 333 K. This range was chosen due to the typical operating conditions of electronic systems, which often reach elevated temperatures during operation due to heat generation in active and passive components. During the tests, resistance values and phase shift angle were measured. Analysis of these parameters allowed for the assessment of the electrical conductivity of the tested structures and the influence of frequency and temperature on their electrical properties. Based on the measured resistance values and the known geometric dimensions of the samples, the electrical conductivity values of the tested layers were calculated. Determining the conductivity enabled a quantitative analysis of electric charge transport in multilayer Ni/SiO_2_ structures. The measurement setup, together with the configuration of the measurement system and a detailed description of the research procedure, is presented in more detail in [15,36].

The electrical properties of the resulting structure were first measured immediately after the layer deposition process. The samples were then annealed in a resistance furnace in an oxygen atmosphere at 400 °C for 30 min. The annealing process was intended to promote partial oxidation of the nickel layers and thereby modify the electrical, magnetic, and structural properties of the multilayer structure. After the annealing process, repeated electrical measurements were performed, allowing for a comparison of the sample properties before and after thermal treatment and an assessment of the effect of the oxidation process on the electric charge transport mechanisms. The 400 °C annealing temperature was chosen based on our previous experimental results and literature reports which indicate that nickel oxidation begins in this temperature range. This temperature was therefore chosen to initiate controlled structural and chemical changes in the nickel-containing layers, including possible partial nickel oxidation, while limiting excessive oxidation and degradation of the multilayer structure. Measurement was repeated 10 times to verify the reproducibility of the results. The measurement conditions were selected to avoid self-heating of the sample. The applied voltage of 1 V does not produce a measurable increase in sample temperature. Therefore, the observed changes in the electrical parameters were not attributed to measurement-induced heating.

### 2.3. Measurements of Magnetic Parameters of Ni-SiO_2_ Nanocomposites

Magnetic fields influence electron transport through spin-related mechanisms. In granular systems, tunneling magnetoresistance (TMR) typically plays a dominant role [37]. Electrons moving between ferromagnetic grains through tunneling retain their spin information. When the magnetic moments of the grains align in parallel under the influence of an external magnetic field, electron spin scattering decreases and the tunneling probability increases. Consequently, the material’s resistance changes. This phenomenon is a classic example of the spintronic effect observed in granular materials [38]. In the case of nickel, anisotropic magnetoresistance (AMR) is a crucial phenomenon [39]. This effect is based on the dependence of electrical resistance on the angle between the direction of current flow and the direction of sample magnetization, as described by Equation (2). Ferromagnetic materials exhibit the AMR effect. This phenomenon results from spin interactions, which influence the scattering of conduction electrons. Depending on the magnetization orientation, electrons undergo different scattering, leading to changes in the material’s resistance. In the presence of a magnetic field, the magnetic moments of nickel grains change their arrangement, which affects electron transport and causes a change in the sample’s resistance. In granular systems, the AMR effect can coexist with TMR, influencing the observed transport characteristics [39,40,41].

(2) $$AMR=\frac{R_{p}-R_{pp}}{R_{0}}$$

where *R_p_* denotes parallel magnetization resistance, *R_pp_* denotes perpendicular magnetization resistance, and *R*_0_ denotes average resistance.

In granular Ni-SiO_2_ structures, conventional AMR associated with spin–orbit-dependent scattering may coexist with tunneling-related contributions. When electron transport includes tunneling between ferromagnetic grains separated by dielectric barriers, an anisotropic tunneling contribution may also occur because the tunneling conductance can depend on the magnetization orientation.

**Figure 2:**
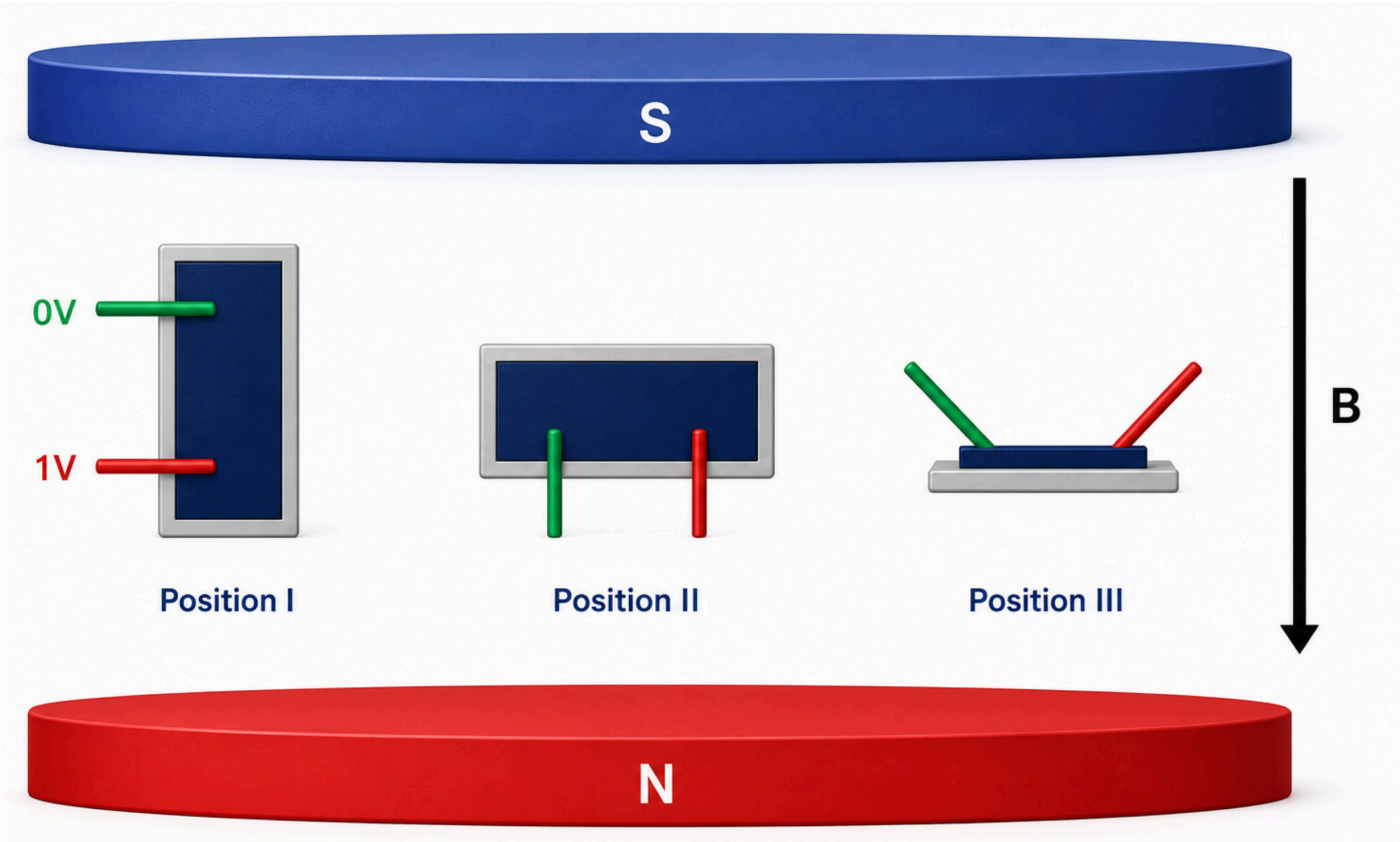
Sample orientations relative to the magnetic field direction, along with the wires leading to the meter, mounted using conductive glue.

Measurements of the magnetoelectric properties of the tested structures were also conducted using a Hioki 3536 RLC meter (Hioki, Nagano, Japan) in the frequency range from 4 Hz to 1 MHz. During the tests, the resistance values of the samples were recorded in the presence and absence of an external magnetic field. Analysis of resistance changes under the influence of the magnetic field enabled the assessment of the influence of magnetic interactions on the transport properties of the multilayer Ni/SiO_2_ structures. Due to instrument limitations related to the ability to precisely adjust the magnetic field, measurements were performed for only two measurement conditions: without an applied magnetic field and in the presence of a 0.5 T magnetic field. The magnetic field was obtained using two neodymium magnets mounted at a constant distance equal to 4 cm from each other, ensuring repeatable measurement conditions for all tested samples. The positions and orientations of the samples relative to the magnetic field direction in which the measurements were performed are shown in Figure 2. The three configurations describe different relative orientations of the current, magnetic field and sample plane. In position I, the current direction is collinear with the applied magnetic field. In position II, the current is perpendicular to the field, while the field remains parallel to the sample plane. In position III, the current is also perpendicular to the field, but the field is normal to the sample plane. If the applied field is sufficiently high to align the magnetization with the field, position I corresponds nominally to the parallel configuration, whereas positions II and III correspond to two perpendicular configurations, namely in-plane and out-of-plane, respectively.

### 2.4. Simulations of Electric and Magnetic Field Distribution

Electric and magnetic field simulations were performed in Sim4Life. First, a model of the n-Si/Ni-SiO_2_ nanocomposite was developed, representing the actual dimensions of the resulting structure, and then appropriate material parameters, including electrical conductivity, were assigned to it. To simulate the electric field, one edge of the structure was assigned a potential of 1 V and the opposite edge was assigned a potential of 0 V. These defined boundary conditions enabled the determination of the electric field distribution. A model corresponding to the actual measurement conditions was developed to simulate the magnetic field. Two cylindrical neodymium magnets, 2 cm in diameter and 1 cm thick, were placed on either side of the structure 4 cm apart. Based on the teslameter measurement, the magnetic field induction between the magnets was determined to be 0.5 T and was adopted as the simulation parameter.

### 2.5. Structural and Chemical Composition Studies of the Ni-SiO_2_ Nanocomposite

The surface morphology and chemical composition of the resulting Ni–SiO_2_ nanocomposite were analyzed using a Zeiss EVO MA10 scanning electron microscope (SEM) (Carl Zeiss, Jena, Germany) equipped with an EDAX Octane Elite APEX EDS energy-dispersive X-ray spectroscopy system (AMETEK, Berwyn, USA). SEM observations were conducted at an accelerating voltage of 5 kV using a secondary electron detector, which enabled detailed analysis of the topography and microstructure of the studied layers. Meanwhile, to confirm the elemental composition, additional EDS analysis was performed at the higher accelerating voltage, adjusted to ensure effective excitation of the characteristic lines of the nickel, silicon and oxygen elements. During the measurements, the electron beam current was increased to 0.5 nA, which allowed for the appropriate number of pulse counts and optimal detector dead time (DT). The working distance was maintained at the recommended level for the EDS detector used, ensuring stable measurement conditions and high quality of the obtained X-ray spectra.

### 2.6. Thickness and Surface Area Studies of Ni-SiO_2_ Nanocomposites

Thickness measurements were performed using a Keyence VK-X3000 series 3D confocal laser microscope (Keyence, Osaka, Japan). The device utilizes triple-scanning technology, including confocal laser measurement, refocusing, and light interferometry, allowing for the selection of the optimal measurement technique based on the characteristics of the object being examined. The microscope provides high-resolution three-dimensional surface measurements and was used in this study to determine the thickness of the deposited layers from the measured height difference between the layer and the substrate. The layer thickness was determined from the difference in height between the coated and uncoated regions of the sample. For each sample, measurements were performed at several locations, and the mean thickness and corresponding standard deviation were calculated.

## 3. Results and Discussion

### 3.1. Measurements of Electrical Parameters and Conduction Mechanism in Ni-SiO_2_ Nanocomposites

**Figure 3:**
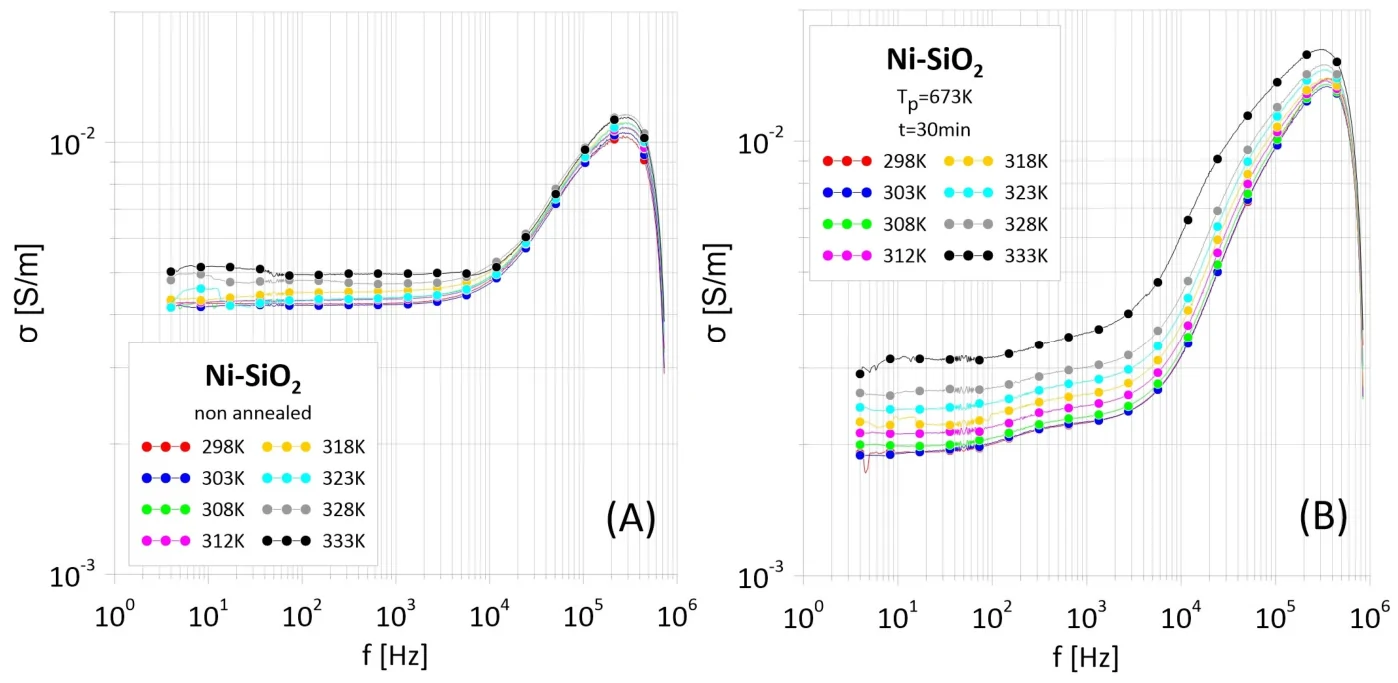
Dependence of conductivity as a function of frequency of the structure before annealing (**A**) and after annealing at 673 K for 30 min (**B**).

Figure 3 presents the relationship between conductivity and frequency for samples before and after the annealing process. Measurements were taken over a temperature range from room temperature to 333 K, with temperature increases in 5 K increments. Measurements were conducted over a wide frequency range from 4 Hz to 1 MHz, allowing for analysis of the electrical response of the samples under both low- and high-signal-frequency conditions. The measurement contacts were placed on the sample surfaces at a constant distance of 5 mm, ensuring repeatable measurement geometry for the sample before and after heating. Measurements were performed in a current-to-plane configuration, enabling recording of the current flowing between the electrode and the sample surface. To minimize the impact of the measurement process on the thermal properties of the material, a constant measurement voltage of 1 V was applied. This voltage value prevented additional heating of the sample during the measurements. Temperature control was performed using a heating table, which allowed for stable annealing of the samples in the measurement chamber. The temperature was monitored and adjusted manually using a thermometer, allowing for continuous monitoring throughout the experiment.

The dependence of conductivity on frequency for the sample before and after annealing at 400 °C is shown above. In both cases, conductivity remains practically constant in the low-frequency range and is independent of frequency. For the sample before annealing, this range covers frequencies up to approximately 10 kHz, while after annealing it shifts to lower values, around 100 Hz.

Above these frequencies, a linear increase in conductivity is observed, characteristic of the electron hopping mechanism described by the Mott model [42]. For the sample immediately after assembling the nanocomposite, the conductivity value changes slightly, but increases with the measurement temperature. This indicates the capacitive nature of the structure, suggesting that the system is close to the percolation threshold but still below its critical value. In the temperature range from 298 K to 323 K, the conductivity values are practically the same and remain constant over most of the frequency range studied. At the same time, the constant nature of conductivity with increasing temperature and frequency is typical of resistive conductivity, confirming the limited change in the charge transport mechanism in this range [15]. After annealing, two characteristic crossover points are visible, the first around 100 Hz and the second above 1 kHz. This may indicate the presence of two different conduction mechanisms characterized by different activation energies. Additionally, after annealing, the conductivity decreases to values more typical of dielectrics, and the differences between the results obtained at different temperatures become significantly greater. This indicates that the annealing process led to oxidation of the nickel layers and grains, which resulted in a shift in the percolation threshold. The decrease in conductivity observed at high frequencies can be attributed to the skin effect. This effect is a side effect and is rather undesirable, as it does not result directly from the conduction mechanism in the material, but rather from the limitation of current penetration into the surface layers of the sample at high frequencies [43,44]. Even when relatively high conductivity and enhanced magnetic permeability were assumed, the calculated skin depth remained much larger than the film thickness. Therefore, the current density can be considered approximately uniform across the layer thickness, and the high-frequency decrease in conductivity cannot reasonably be attributed to the classical skin effect.

The decrease observed near the upper limit of the investigated frequency range may instead be associated with the parasitic capacitance or inductance of the leads and sample holder, contact impedance, limitations of the equivalent-circuit representation, or the frequency response of the measurement system. Because these contributions were not independently separated, the high-frequency decrease is not assigned to a specific intrinsic transport mechanism and was excluded from the Jonscher law fitting. Throughout the entire frequency range analyzed, DC conduction was initially observed, followed by a gradual increase in electrical conductivity σ and a simultaneous decrease in resistance *Rp*, consistent with Jonscher’s law [45].

The frequency dependence of the real conductivity was quantitatively analyzed using Jonscher’s power law:

(3) $$\sigma \left(f\right)=\sigma_{dc}+{A(2\pi f)}^{s}$$

where $\sigma_{dc}$ represents the frequency-independent conductivity, A is the coefficient of the dispersive contribution, and s is the frequency exponent.

The fitting was performed in the range from 4 Hz to approximately 150 kHz, covering the low-frequency conductivity plateau and the dispersive increase. The high-frequency conductivity maximum and the subsequent decrease were excluded because they cannot be described by the monotonically increasing Jonscher relation. For the non-annealed structure, the fitted exponent ranged from 0.732 to 0.943, while the coefficients of determination ranged from 0.977 to 0.990. For the annealed structure, s decreased from 0.667 at 298 K to 0.489 at 333 K, with *R*^2^ values between 0.982 and 0.992. These results confirm that Jonscher’s relation provides a good quantitative description of the conductivity plateau and the dispersive region for both structures. However, the different temperature evolution of *s* indicates that annealing modified the frequency-dependent charge-transport response. The *σ_dc_* values obtained from the Jonscher fits were subsequently used to evaluate the temperature dependence of conductivity. In the non-annealed structure, σdc increased from approximately 4 × 10^−3^ to 5 × 10^−3^ S/m, between 298 and 333 K. After annealing, the conductivity was lower and increased from approximately 1.9 × 10^−3^ to 2.75 × 10^−3^ S/m. The stronger relative temperature dependence after annealing suggests an increase in the effective energy barriers controlling charge transport.

The temperature dependence was tested using the Arrhenius and variable-range hopping representations. The Arrhenius relation was expressed as

(4) $$\sigma_{dc}\left(T\right)=\sigma_{0}exp\left(-\frac{E_{a}}{k_{B}T}\right)$$

where $\sigma_{0}$ is the pre-exponential factor and corresponds to the theoretical value of conductivity at very high temperatures, $E_{a}$ is activation energy of the conduction process, and $k_{B}$ is the Boltzmann constant.

In contrast, the three-dimensional Mott VRH model was written as

(5) $$\sigma_{dc}\left(T\right)=\sigma_{0}exp\left[-{\left(\frac{T_{0}}{T}\right)}^{1/4}\right]$$

Here, $T_{0}$ is the characteristic Mott temperature and is related to the density of localized states at the Fermi level by

(6) $$T_{0}=\frac{\beta }{k_{B}N(E_{F})\xi^{3}}$$

where β is a model-dependent coefficient, $N\left(E_{F}\right)$ is the density of localized states at the Fermi level, and ξ is the localization length.

Annealing therefore reduced the absolute conductivity and increased the apparent activation energy, indicating that the charge carriers encountered higher effective transport barriers after thermal treatment. This behavior may result from changes in the microstructure, intergranular regions, metal–dielectric interfaces, or the degree of carrier localization.

**Figure 4:**
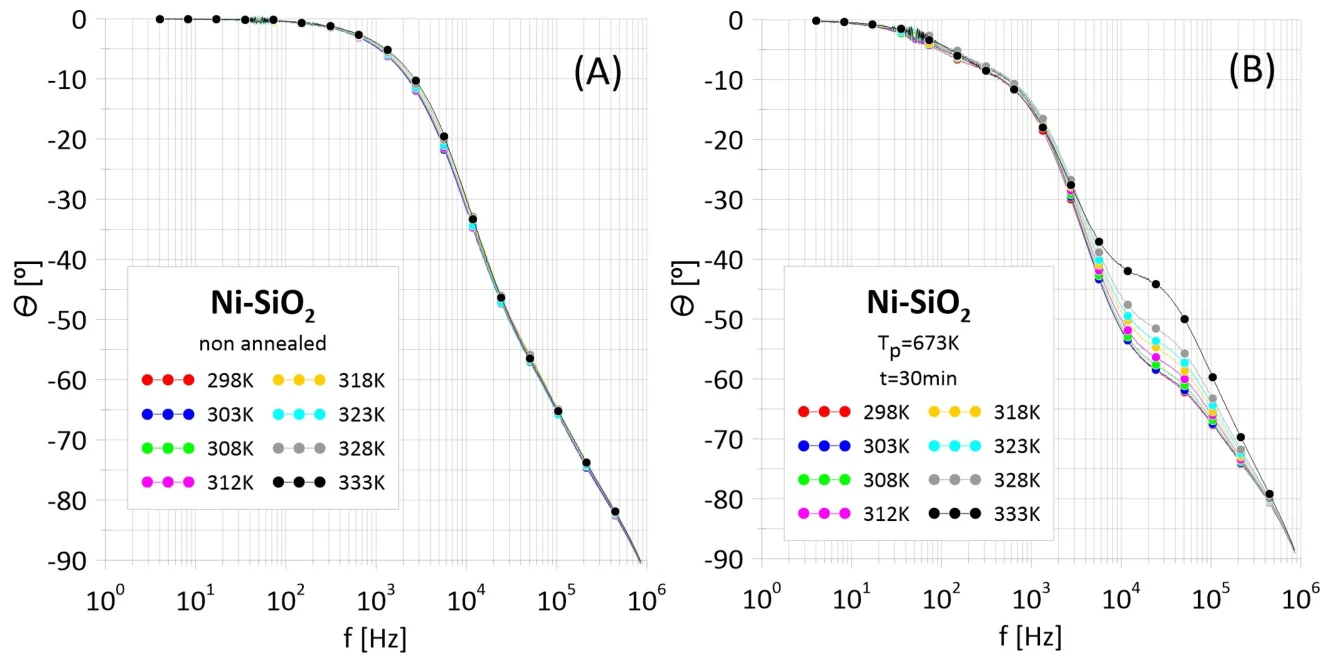
Dependence of phase shift angle as a function of frequency of the structure before annealing (**A**) and after annealing at 673 K for 30 min (**B**).

These phenomena indicate the gradual formation of effective charge transport pathways. The annealing process increases the average distance between active centers, which consequently impedes the movement of charge carriers. In the described phenomenon, ions originating from the dielectric matrix and the nanoparticle surface accumulate at the phase boundary, forming an electrical double layer. In the annealed sample the temperature could have caused the nanoparticles to organize, which may also contribute to this process. This layer acts as a capacitor with very high capacitance, effectively limiting the flow of direct current and low-frequency currents. A negative phase angle (Figure 4) indicates the capacitive nature of the impedance in this region where the current leads the voltage.

Figure 4 shows the phase angle versus frequency for the sample before and after thermal processing. This relationship allows for a comparison of changes in the material’s electrical properties induced by thermal processing over a wide range of signal frequencies. Based on the obtained results, it can be observed that in both analyzed cases, the phase angle changes monotonically, ranging from approximately 0° at low frequencies to approximately −90° at high frequencies. This pattern of change is typical for systems with behavior corresponding to a parallel connection of resistive and capacitive elements. The observed waveform indicates that the dominant conduction mechanism in the tested samples can be described by a parallel RC circuit model, in which the influence of the capacitive component on the overall system response increases with increasing frequency. The observed inflection in the frequency range of 10^4^–10^5^ Hz after annealing indicates the coexistence of at least two relaxation processes with similar time constants. In the case of the Ni–SiO_2_ nanocomposite, with its highly heterogeneous electrical structure, this effect results from the overlap of relaxation within the metallic grains and Maxwell–Wagner–Sillars (MWS) interfacial polarization, associated with charge accumulation at the metal–dielectric interfaces. The more pronounced appearance of this phenomenon at higher temperatures, particularly at 333 K, indicates its thermally activated nature. Different activation energies cause different changes in time constants with temperature, leading to the separation of components in the frequency domain and the unmasking of a previously masked effect. Asymmetries and phase angle patterns are typical of heterogeneous materials and indicate the presence of structures characterized by series-connected RC elements. Therefore, in this case, the system can be considered not only as a resistor connected in parallel with a capacitor, but also as a series RC circuit [46,47].

**Figure 5:**
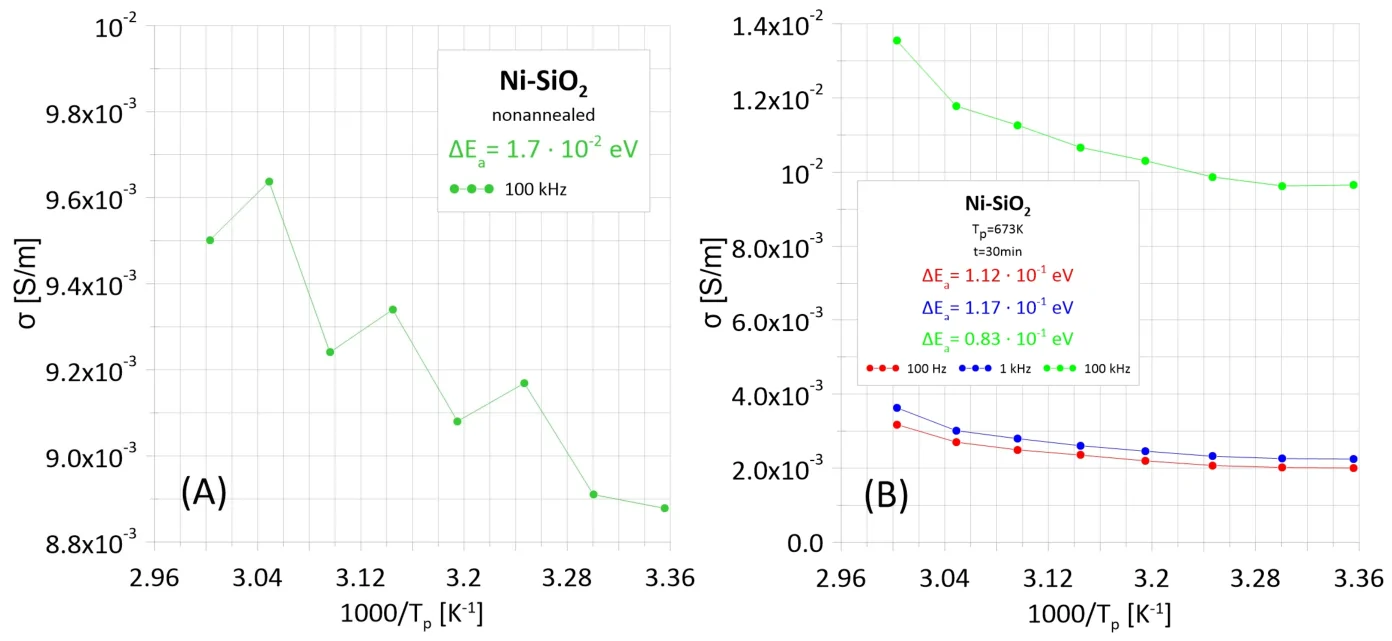
Arrhenius relationship for structure before annealing (**A**) and after annealing at 673 K for 30 min (**B**).

The Arrhenius relations, shown in Figure 5, were determined for both analyzed samples before and after the annealing process. Analysis of the characteristic curves reveals significant differences in the charge transport mechanisms occurring in both systems [48]. In the case of the sample before annealing, the graph does not exhibit a clearly linear character, but rather a downward trend, indicating the presence of a thermally activated conduction mechanism described by the Arrhenius equation. At the same time, the visible local decreases and increases in conductivity values suggest instability in the conduction process. This may indicate that the system under study is near the percolation threshold, where even small changes in energy or temperature cause the formation and dissolution of conductive paths [49]. In this case, charge transport occurs primarily through electron hopping between local conduction centers. The determined activation energy for the unannealed sample is 1.7 × 10^−2^ eV, indicating that the conduction process requires relatively little energy for a single electron to jump. A similar general pattern is also observed for the sample after annealing, but in this case, the graph is much more linear. This may indicate greater ordering of the material structure and stabilization of the conduction paths after heat treatment. At the same time, two distinct inflections are visible in the graph, suggesting the presence of a hopping conduction mechanism involving two different charge transport processes characterized by different activation energies [50]. For the first mechanism, dominant at a frequency of 100 Hz, the activation energy is 1.12 × 10^−1^ eV. For the second mechanism, observed at a frequency of 1 kHz, the energy required for electron hopping reaches 1.17 × 10^−1^ eV. Higher activation energy values after annealing may indicate that the heat treatment process has led to a greater distance between active centers or a reduction in the number of available conduction paths, which hinders charge carrier transport and requires a higher energy input for conduction [51,52,53]. After the annealing process, the activation energy determined at a frequency of 100 kHz was 0.93 × 10^−1^ eV. This value was significantly lower than for the unannealed structure measured at the same frequency. The reduction in activation energy may indicate a reduction in the energy barrier associated with carrier transport after annealing. This may be due to a reorganization of the material’s microstructure, a change in the number and arrangement of trap states, or improved connections between conductive regions, which facilitates charge transport.

Nevertheless, the quality of the 3D Mott fit was only slightly better than that of the Arrhenius model. Moreover, the 2D Mott and Efros–Shklovskii representations produced similarly high R^2^ values, particularly for the annealed structure. Therefore, within the limited temperature range of 298–333 K, the results support thermally activated hopping-like transport but do not allow the microscopic conduction mechanism to be assigned unambiguously to the 3D Mott VRH model.

### 3.2. Measurements of the Magnetoelectric Properties in Ni-SiO_2_ Nanocomposites

**Figure 6:**
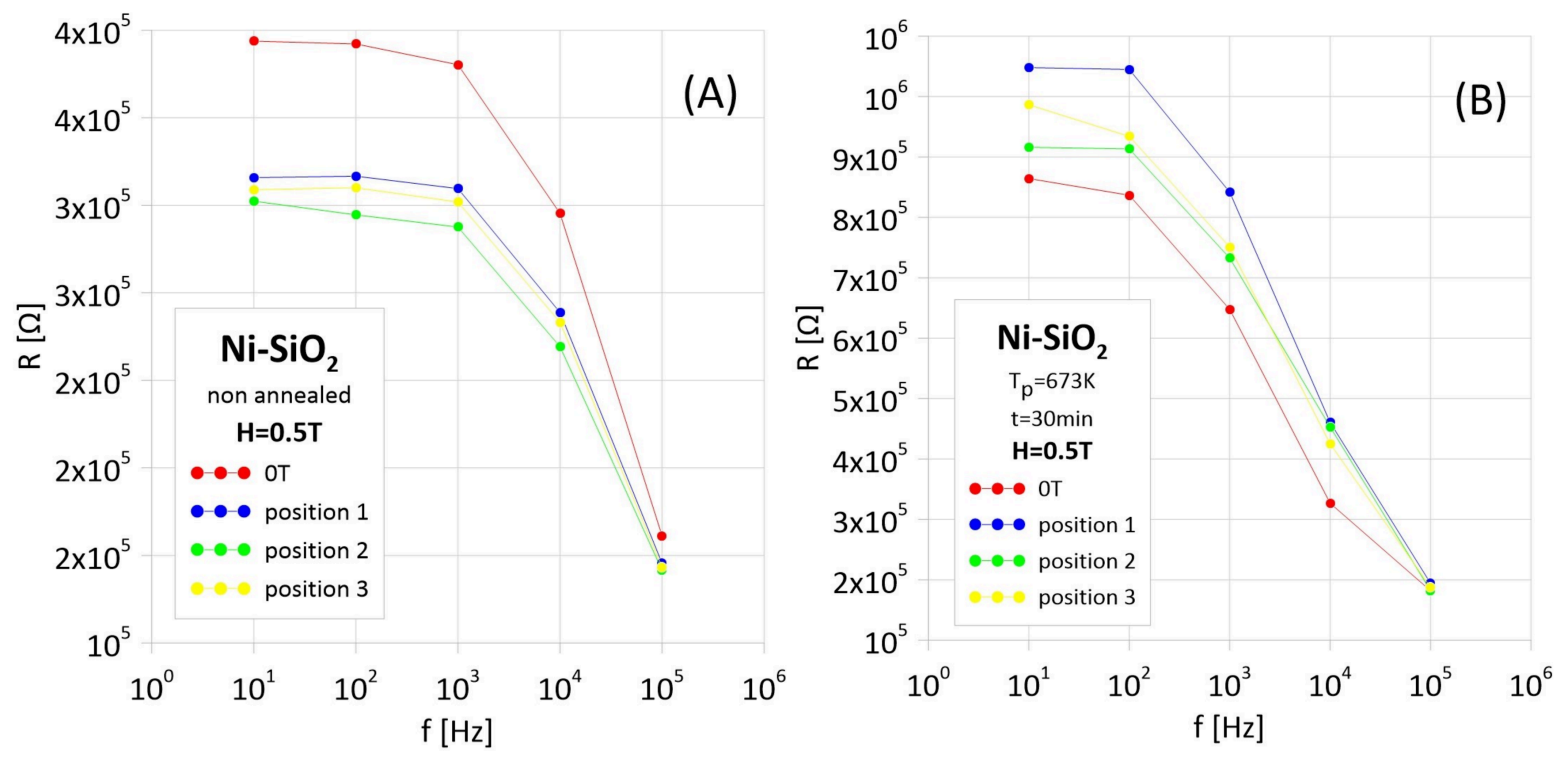
Dependence of resistance as a function of frequency of the structure before annealing (**A**) and after annealing at 673 K for 30 min (**B**) without a field and in a magnetic field in three different orientations.

The graphs presented in Figure 6 illustrate the dependence of resistance on frequency for the Ni–SiO_2_ structure tested in a magnetic field equal to 0.5 T. The graphs shown in Figure 6A and Figure 6B correspond to the sample before and after annealing at 673 K for 30 min, respectively. The measurements were carried out for three different sample positions relative to the magnetic field and for the reference measurement of 0 T. In all analyzed cases, a decrease in resistance is observed with increasing frequency. For the unannealed sample, the presence of a magnetic field causes a decrease in resistance regardless of the sample position. In turn, after annealing, an inverse relationship was observed: the resistance in the presence of a magnetic field reaches higher values than in the reference measurement.

In both graphs, it can be seen that the resistance reaches its highest values at low frequencies and then decreases in the range from 10^4^ to 10^5^ Hz. This behavior is characteristic of relaxation hopping transport, Maxwell–Wagner intergranular polarization, and AC conduction in heterogeneous systems. At low frequencies, charge carriers are strongly affected by the insulating SiO barriers, which leads to an increase in the system resistance. As frequency increases, electrons can tunnel through shorter conduction paths, and the share of slow relaxation processes gradually decreases. At the same time, the importance of capacitive coupling between the material’s grains increases. As a result, a decrease in impedance and resistance is observed at higher frequencies. This type of behavior is typical for granular composites and thin-film oxide systems [44,54]. The most pronounced effect visible in the graphs is the significant increase in the sample’s resistance after the annealing process. The resistance value increases by more than one order of magnitude compared to the unannealed sample. This may indicate the simultaneous occurrence of several physical processes in the material. One of the most likely mechanisms is the partial oxidation of nickel during annealing. Under such conditions, some of the metallic Ni may transform into nickel oxide, NiO. Because NiO has a significantly lower electrical conductivity than metallic nickel, this leads to an increase in the tunnel barrier height between grains, a decrease in the concentration of free electrons, and an increase in the resistance of the entire system. Such phenomena have been previously observed in NiO/SiO_2_ nanostructures [55,56]. The annealing process leads to a number of structural changes in the material. It can cause coalescence of nanoparticles, a change in grain size, reorganization of percolation pathways, and diffusion of Ni atoms in the SiO_2_ matrix. These changes directly affect the electrical and magnetic properties of the system being studied. The annealing process leads to an increase in nanoparticle size and significant changes in the magnetic properties of the material. The system can also transition from a nonmagnetic to a magnetic phase [55,57]. In the sample after annealing, the differences between the individual measurement series are clearly greater than before annealing. This may indicate an increase in local magnetic inhomogeneity, the appearance of stronger tunneling barriers, and a greater contribution of electron spin-dependent transport. The annealing process could have caused a change in the nature of electron transport from a more metallic to a more hopping mode. In this case, electrons move primarily by tunneling between ferromagnetic grains, which increases the influence of magnetoresistance effects. For this reason, an increase in the magnetoresistance of the system is often observed after annealing. For frequencies around 10^5^ Hz, all curves approach very similar resistance values. This indicates that at such high frequencies, the transport mechanism changes. In this frequency range, the capacitive response of the system begins to dominate, while the importance of local potential barriers and the structure of magnetic domains decrease significantly. As a result, the system is no longer accurately described as a tunnel network with clearly defined obstacles to electron flow. Instead, it behaves more like an effective RC circuit, in which resistive and capacitive elements become crucial than tunneling mechanisms.

Table 1 shows the magnetoresistivity coefficient *ΔR*/*R* values of the structure before and after annealing. For the sample before annealing, the *ΔR*/*R* has exclusively negative values, on the order of 15% to 20% at low frequencies, decreasing to approximately 7% at 100 kHz. This indicates that the magnetic field lowers the system resistance. The alignment of the magnetic moments in the field reduces electron scattering, leading to a decrease in resistance [58,59]. After annealing, the sign of the effect changes to positive (ca. 6–41%), approaching zero only at 100 kHz. This indicates a transition to a different transport mechanism, one that is more strongly governed by tunneling and barrier-based processes. Annealing increases the barriers, which favors hopping transport and tunneling effects, leading to positive magnetoresistance [60,61]. The strong frequency dependence indicates that at low frequencies, spin and interface processes dominate, while at high frequencies, the contribution of tunneling decreases due to Maxwell–Wagner relaxation and dominance of the capacitive response. Additionally, after annealing, greater spatial anisotropy appears. Differences between measurement positions are clear, indicating a heterogeneous structure and local percolation changes in the system.

The reversal of the sign of *ΔR* after annealing shows that the thermal treatment modified the field-sensitive electrical response of the nanocomposite. This change may be associated with annealing-induced modifications of the metallic-grain arrangement, intergranular barriers, metal–dielectric interfaces, magnetic-domain configuration, or the relative contribution of different conduction paths.

However, the present measurements were performed only at 0 T and at a single positive field value of approximately 0.5 T. Therefore, they do not provide information about the full *R*(*H*) dependence, field symmetry, coercive effects, hysteresis or measurement-cycle repeatability. Consequently, the observed sign reversal cannot be considered direct evidence of a transition from spin-dependent scattering to spin-dependent tunneling. It should instead be interpreted as a phenomenological indication that annealing changed the dominant field-sensitive contribution to electrical transport.

### 3.3. Simulations of Electric and Magnetic Properties of Ni-SiO_2_ Nanocomposites

**Figure 7:**
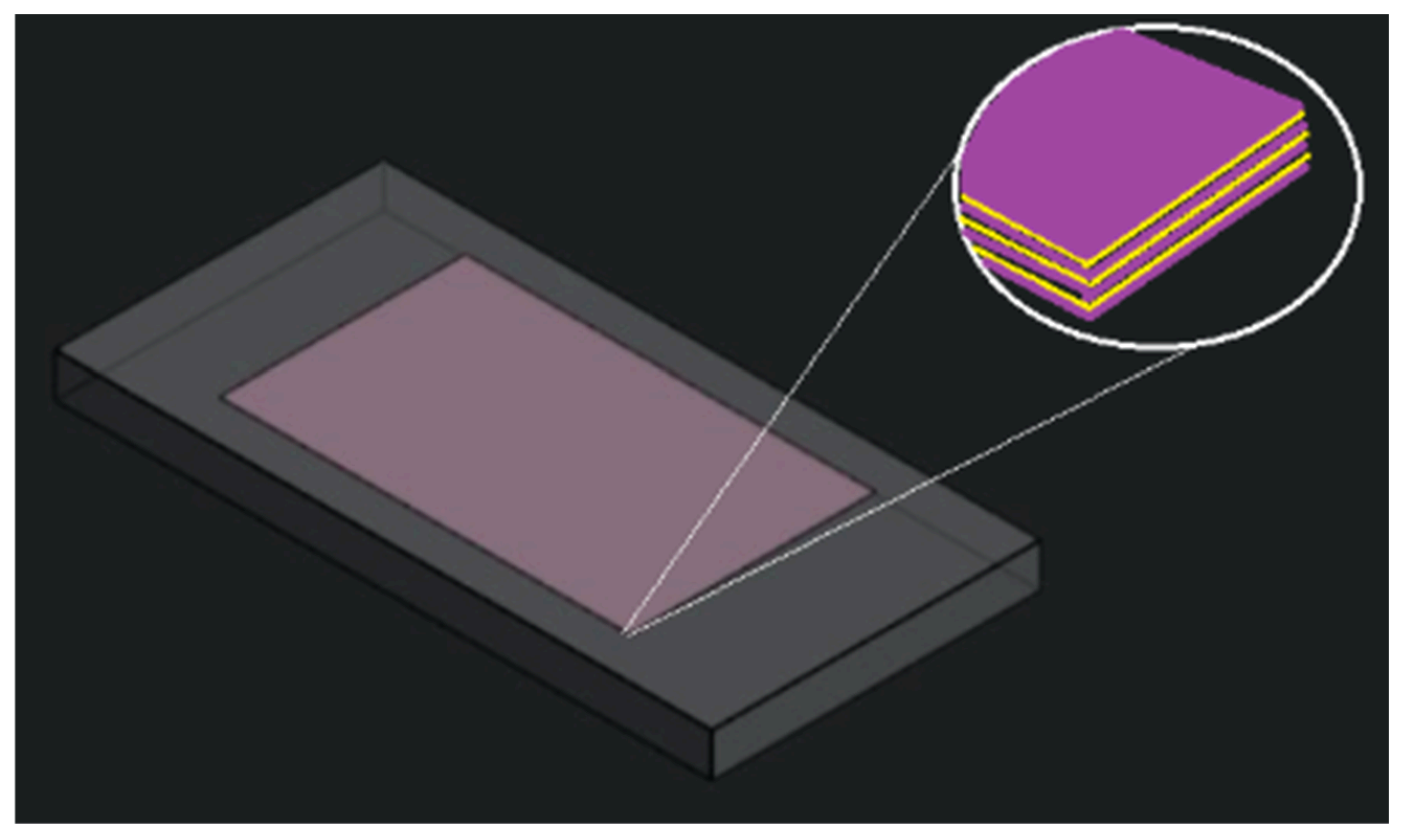
Ni-SiO_2_ nanocomposite structure designed for simulation. SiO_2_ is shown in yellow, and Ni in purple.

As part of the numerical calculations performed in the Sim4Life Lite (version 8.2 for student) environment, a model of the nSi/Ni-SiO_2_ nanocomposite was developed, representing the actual dimensions of the resulting structure, and then its material properties, including electrical conductivity, were defined. Figure 7 shows the structure designed in the program, where SiO_2_ layers are marked in yellow and Ni layers in purple. The gray rectangle around the structure is the n-Si substrate.

**Figure 8:**
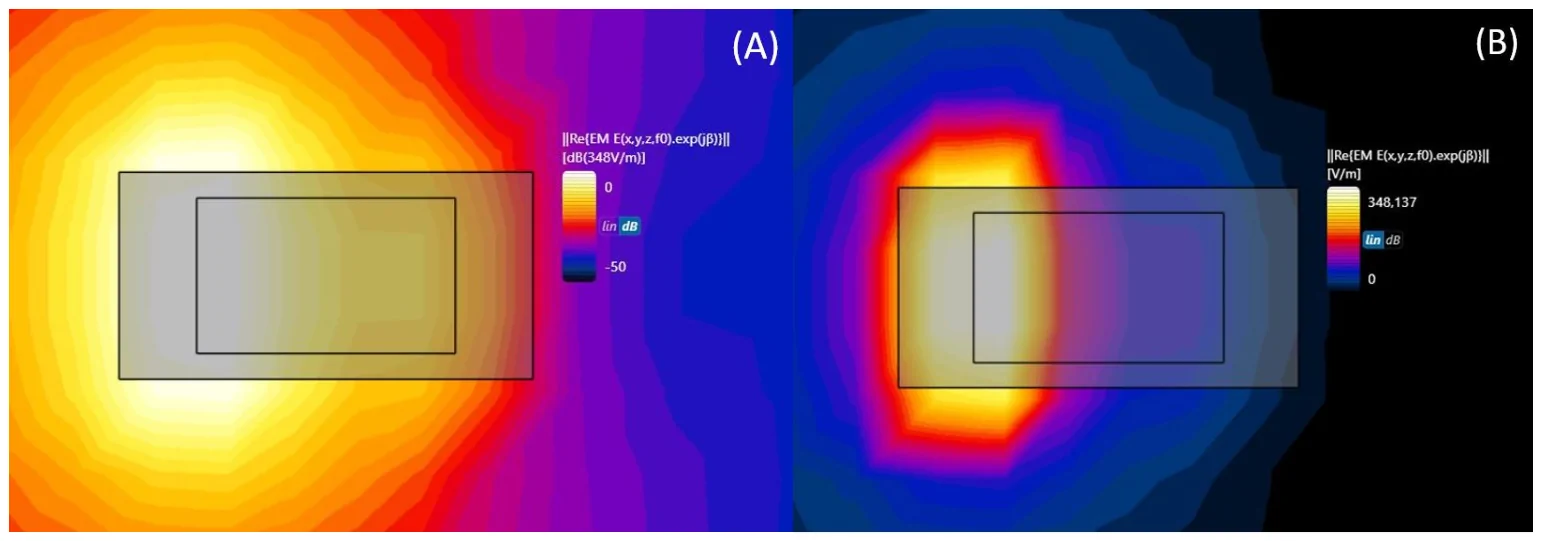
Electric field simulation of the Ni-SiO_2_ nanocomposite in dB (**A**) and linear (**B**) scales.

To simulate the electric field, potentials of 1 V and 0 V were applied to opposite edges of the structure at 100 Hz. Figure 8A shows the field intensity on a linear scale, with the highest values near the 1 V boundary and a gradual decrease toward the grounded edge. Local variations reflect the influence of the internal geometry. Figure 8B presents the same distribution on a logarithmic scale, highlighting the field gradient over a wider dynamic range.

**Figure 9:**
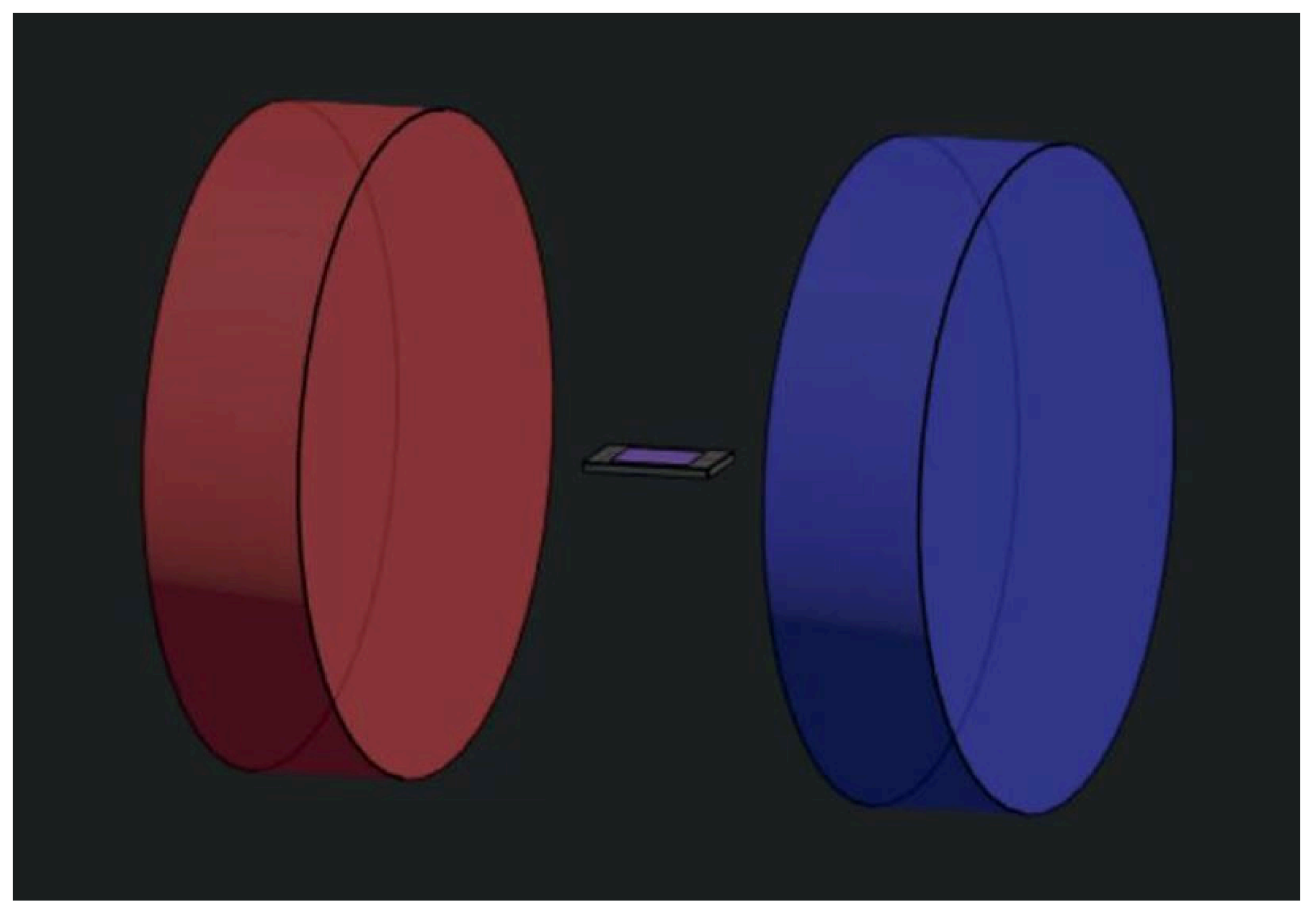
Designed structure of a Ni-SiO_2_ nanocomposite with neodymium magnets.

To simulate the magnetic field, a model reflecting actual measurement conditions was designed and is shown in Figure 9. Two cylindrical neodymium magnets were placed on either side of the structure, 4 cm apart; each magnet was 2 cm in diameter, and 1 cm thick. Based on measurements taken with a teslameter, the magnetic field induction value in the space between the magnets was determined to be 0.5 T, and this parameter was adopted for the simulation.

**Figure 10:**
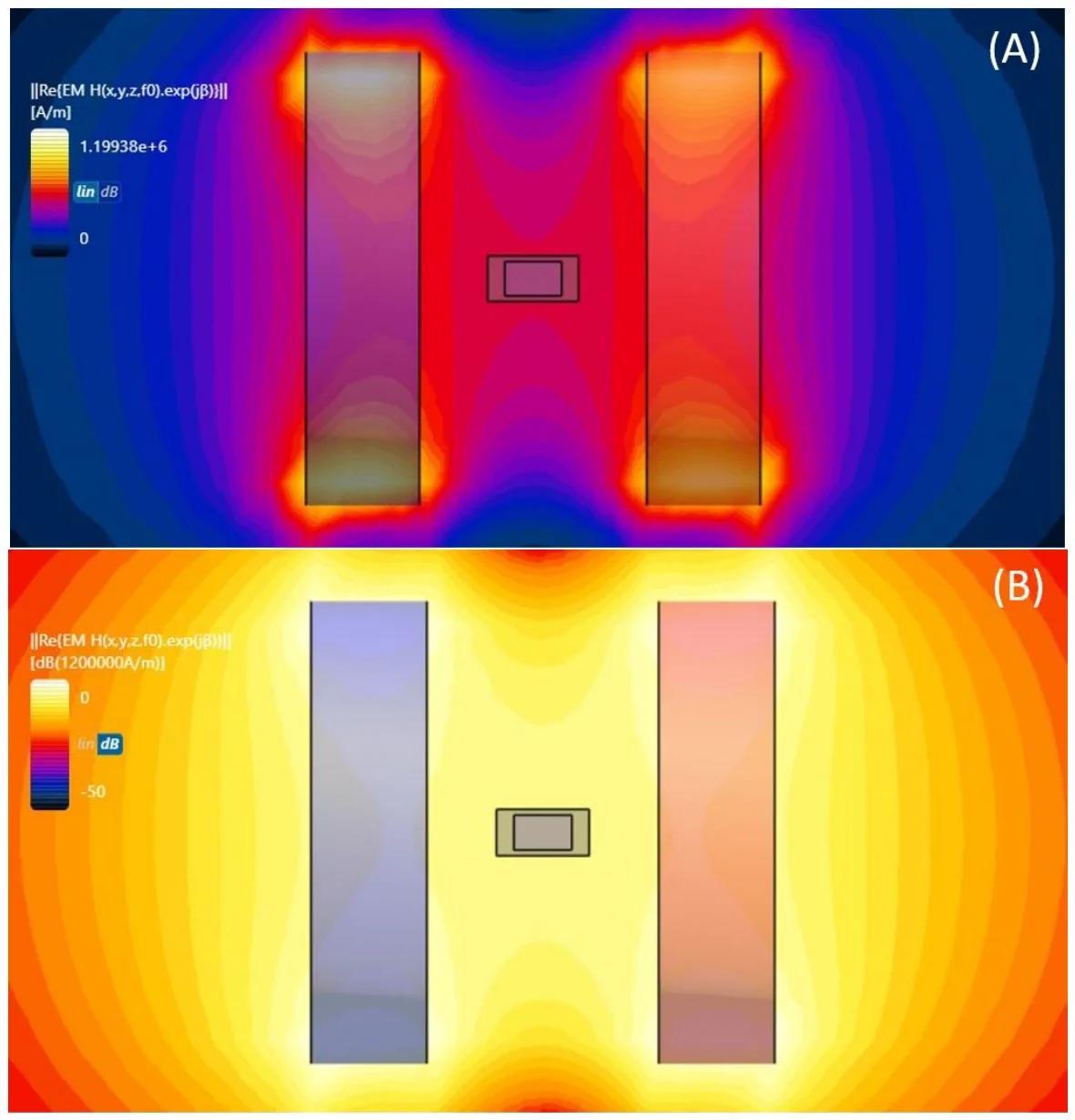
Magnetic field simulation of the Ni-SiO_2_ nanocomposite in dB (**A**) and linear (**B**) scales.

Figure 10A presents the magnetic field intensity on a linear scale. The highest values occur near the edges and corners of the two vertical conductive elements, where the field lines are concentrated. In the region between the conductors, including the centrally placed element, the field distribution is more uniform and gradually decreases with distance from the active area. Figure 10B shows the same distribution on a logarithmic scale, enabling weaker field variations to be observed more clearly. The map confirms the approximately symmetrical field distribution and its rapid attenuation outside the excitation region. Overall, the simulation indicates that the central part of the structure was exposed to a relatively uniform magnetic field during the measurements.

### 3.4. Structural and Chemical Composition Analysis of Ni-SiO_2_ Nanocomposites

**Figure 11:**
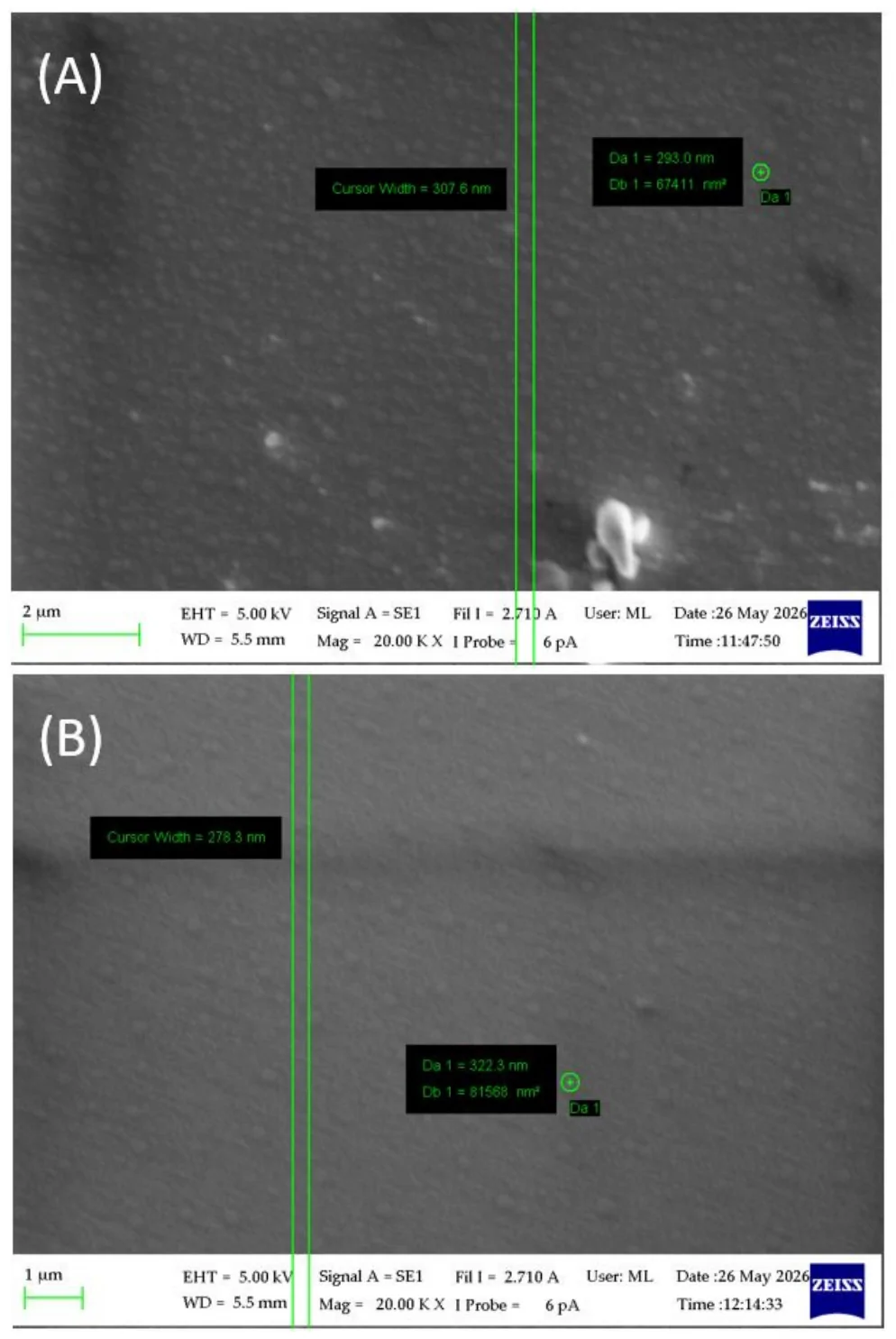
SEM image of the nanocomposite Ni-SiO_2_ before (**A**) and after annealing (**B**).

Figure 11 presents SEM images of the structures before and after the annealing process. Both images show a non-uniform surface morphology with quite distinguishable island-like features, whose representative lateral dimensions are approximately 280–320 nm, as indicated directly in the micrographs. Similar observations have been made based on studies of similar granular structures [62]. The observed morphology is therefore consistent with the assumption that the nominally 1 nm thick Ni and SiO_2_ deposition increments did not form an ideal stack of fully continuous films but developed into larger island-like regions, leading to the formation of a granular nanocomposite structure. This type of behavior is characteristic of ultrathin metallic and dielectric layers, for which the surface energy and interfacial interactions favor discontinuous growth [36,63]. Analysis of the SEM images also indicates that the annealing process did not significantly increase grain size. However, a significant increase in the distance between grains after annealing can be observed, which may indicate partial structural reorganization and dewetting processes leading to the separation of material islands [64,65]. This phenomenon is often observed in ultrathin films deposited on SiO_2_ substrates, where annealing minimizes the surface energy of the system and increases the separation between material grains or islands [66,67].

Table 2 presents the EDS analysis results for the structures before and after annealing. The results indicate that the annealing process did not significantly affect the overall elemental composition of the tested structures within the accuracy of the EDS method. In both cases, the oxygen content remained virtually unchanged; before annealing, the oxygen weight fraction was 6%, while after annealing it was 6.1%. Similarly, the atomic fraction changed only from 10.6% to 10.8%. Such small differences are within the typical measurement uncertainty of the EDS method and do not indicate an intensification of the oxidation process during annealing. The silicon content also remained virtually unchanged. The weight fraction decreased only from 85.1% to 84.4%, while the atomic fraction decreased from 85.2% to 84.6%. The high silicon content recorded in the EDS analysis results not only from the presence of the SiO_2_ in the nanocomposite structure, but also from the contribution of the silicon substrate to the measured signal. The slight increase in nickel content after annealing, from 8.9% to 9.5% by weight and from 4.3% to 4.6% by atom, may result from local compositional heterogeneity, differences in the analyzed area, or surface morphological changes observed in the SEM images. The annealing conditions used were insufficient to induce significant oxygen diffusion or oxide layer growth. Moderate annealing leads primarily to morphological changes but does not significantly increase the oxidation of the material, especially in the presence of a stable SiO_2_ substrate [68,69,70].

### 3.5. Surface Area Analysis of Ni-SiO_2_ Nanocomposites

**Figure 12:**
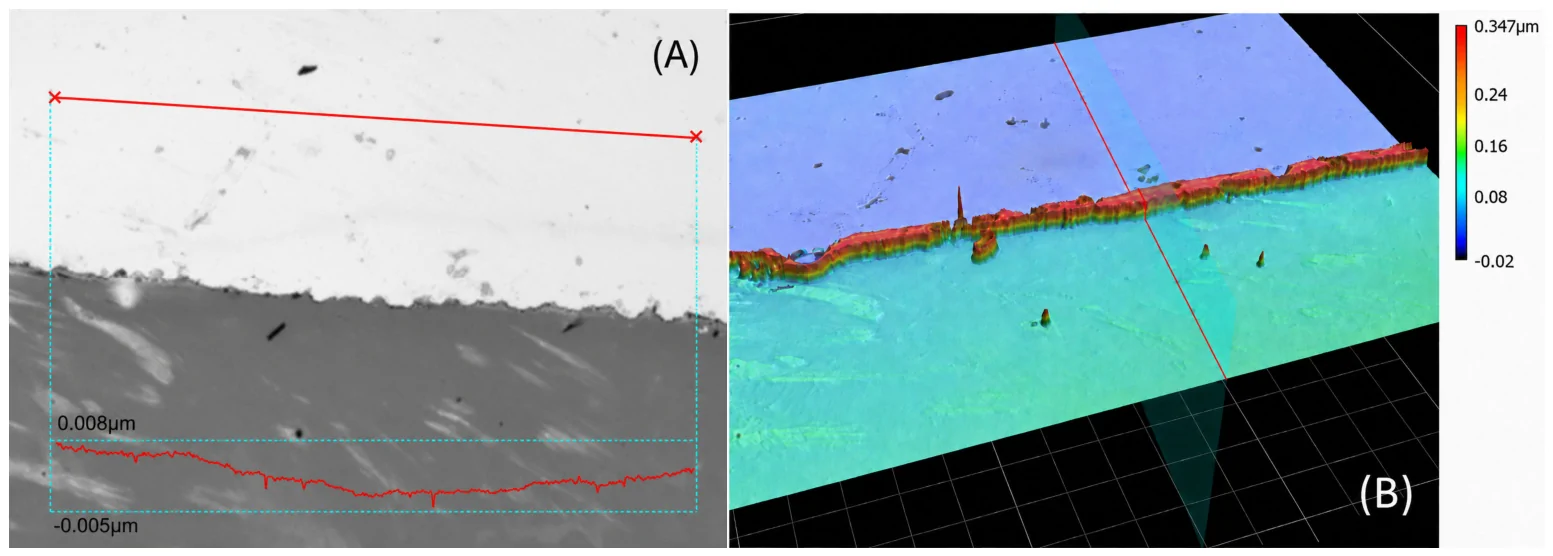
Surface image showing the analyzed regions of the multilayer and the exposed silicon substrate (**A**) and corresponding 3D surface profile obtained using white light interferometry (**B**).

**Figure 13:**
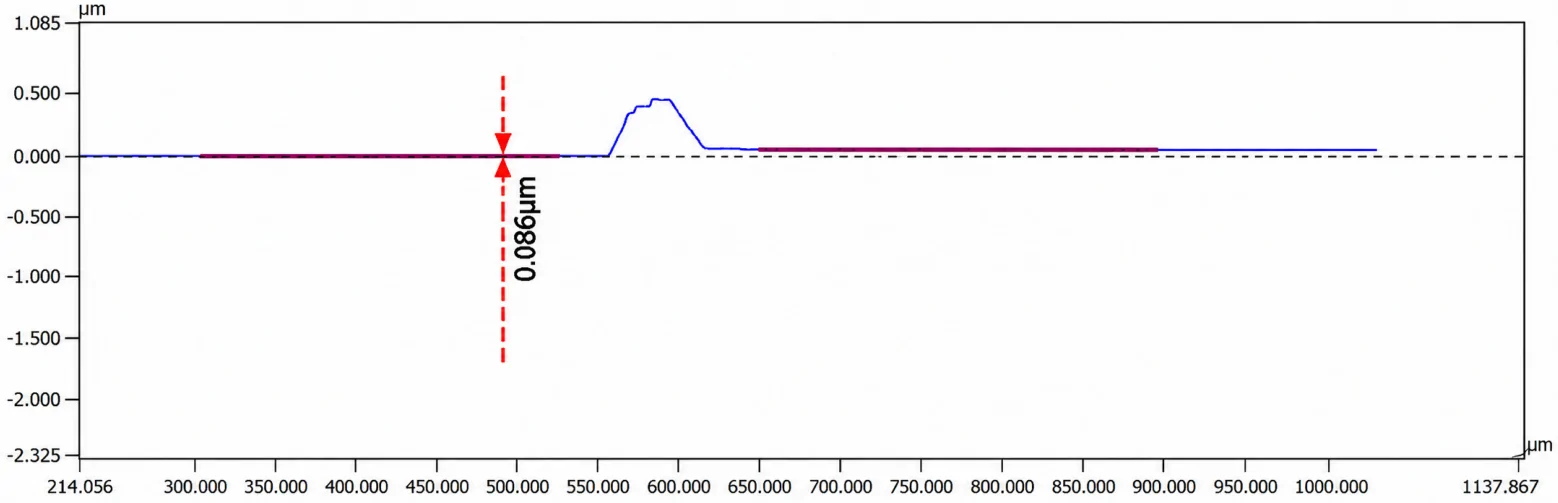
Representative height profile used to determine the thickness of the Ni/SiO_2_ multilayer.

Surface roughness and thickness measurements were performed using the WLI (white light interferometry) method. Figure 12A,B present a top-down image of the surface and a 3D profile of the analyzed structure. The 3D image shows a smooth, linear transition zone between the layer and the substrate, without visible large defects or abrupt changes in surface height. Figure 12A indicates very low surface roughness with the *R*_a_ and *R*_z_ parameters reported by the WLI system as 0 µm. On the other hand, Figure 12B shows a 3D profile with a clear height jump between the layer and the substrate. For the representative measurement shown in Figure 12, the height difference between the layer and the silicon substrate was 0.104 µm. The difference between the highest and lowest points within the analyzed area was 0.531 µm, indicating slight local variations in the surface profile. Figure 13 shows the profile measurement that resulted in a thickness of 86 nm.

WLI analysis revealed that the fabricated Ni/SiO_2_ multilayer exhibited continuous surface coverage without visible cracks or delamination. The thickness of the deposited structure was measured at five different locations across the sample and ranged from 86 to 104 nm, with an average thickness of 95.4 ± 6.8 nm. Although the overall uniformity was satisfactory, local interfacial irregularities and nanoscale protrusions were observed. Considering that the individual SiO_2_ and Ni sublayers had nominal thicknesses of approximately 1 nm, as measured by the silicon sensor inside the sputtering machine; the measured topographical variations indicate relatively high interface roughness with respect to the thickness of the individual layers. This behavior is likely associated with the island-like growth of ultrathin Ni layers and roughness propagation during sequential magnetron sputtering deposition.

## 4. Conclusions

The conducted studies demonstrated that the annealing process significantly affects the electrical and magnetoresistive properties, as well as the microstructure of the Ni–SiO_2_ nanocomposite. Electrical conductivity analysis confirmed that the dominant charge transport mechanism in the studied structures was the hopping mechanism. Before annealing, the material was near the percolation threshold, resulting in relatively high conductivity and a slight temperature dependence of electrical parameters. After the annealing process, a significant decrease in conductivity and an increase in the conduction activation energy were observed, indicating an increase in the distance between active conduction centers and a reduction in the number of charge transport paths.

Phase shift analysis confirmed that the studied system exhibited a capacitive-resistive nature, which can be described by a parallel RC circuit model with additional series RC elements. After annealing, additional relaxation processes associated with Maxwell–Wagner–Sillars interfacial polarization and intragrain relaxation were revealed, indicating increased electrical heterogeneity of the material.

Before annealing, the application of a 0.5 T magnetic field resulted in a decrease in resistance for all three sample orientations. After annealing, an increase in resistance was observed in the low- and intermediate-frequency ranges, while the field-induced differences diminished at higher frequencies. This sign reversal indicates that annealing modified the field-sensitive electrical response of the structure. Nevertheless, because the measurements were limited to B = 0 and one positive field value, the results do not allow the microscopic origin of this behavior to be identified unambiguously. In particular, a transition from spin-dependent scattering to spin-dependent tunneling cannot be confirmed without field-sweep measurements, negative-field data and repeated magnetic-field cycles.

The conducted electric and magnetic field simulations demonstrated a significant influence of the geometry of the analyzed structure and the specified boundary conditions on the electromagnetic field distribution. In the case of the electric field, a distinct potential gradient and intensity concentration were observed near the excitation areas and the edges of the conductive elements. The magnetic field simulation confirmed local field concentration near the conductors and its gradual attenuation with increasing distance from the excitation source. The obtained results indicate that the arrangement of conductive elements and their geometry are crucial for the uniformity and intensity of the field distribution, which is a crucial aspect in the design and optimization of electromagnetic structures.

SEM observations confirmed the granular morphology of the Ni–SiO_2_ layers and the lack of continuous layer formation with a nominal sublayer thickness of 1 nm. The annealing process did not significantly increase grain size, but it did lead to an increase in the distance between grains, which can be attributed to dewetting and structural reorganization. The EDS analysis results showed that the annealing conditions used did not significantly increase the oxidation state of the material, and the observed changes in electrical properties resulted primarily from morphological reorganization and changes in the conductive structure.

The WLI analysis revealed that the obtained structures were characterized by good surface uniformity and low roughness, despite the presence of local irregularities related to the island-like growth of ultrathin nickel layers. The obtained results confirm that Ni–SiO_2_ nanocomposites exhibit a strong dependence of their transport and magnetic properties on heat treatment processes, making them promising materials for applications in magnetoelectric and nanoelectronic systems.

## Figures and Tables

**Table 1:** *ΔR*/*R* values of the structure before and after annealing.

Structure	f [Hz]	Position 1 [%]	Position 2 [%]	Position 3 [%]
Non-annealed	1	−15.80	−18.52	−17.19
	100	−15.35	−19.84	−16.69
	1 k	−14.74	−19.31	−16.34
	10 k	−14.32	−19.26	−15.73
	100 k	−7.38	−9.30	−8.39
Annealed	1	21.25	5.99	14.13
	100	24.89	9.17	11.65
	1 k	29.97	13.13	15.93
	10 k	40.90	38.66	30.09
	100 k	6.73	0.09	3.19

**Table 2:** EDS analysis of the Ni-SiO2 nanocomposite.

Structure	Element	Weight [%]	Atomic [%]
Non-annealing	O	6	10.6
	Si	85.1	85.2
	Ni	8.9	4.3
Annealing	O	6.1	10.8
	Si	84.4	84.6
	Ni	9.5	4.6

## Data Availability

The original contributions presented in this study are included in the article material. Further inquiries can be directed to the corresponding author.
